# A novel web-based dynamic nomogram for recurrent laryngeal nerve lymph node metastasis in esophageal squamous cell carcinoma

**DOI:** 10.3389/fsurg.2022.898705

**Published:** 2022-08-23

**Authors:** Ting-Ting Chen, Hao-Ji Yan, Xi He, Si-Yi Fu, Sheng-Xuan Zhang, Wan Yang, Yu-Jie Zuo, Hong-Tao Tang, Jun-Jie Yang, Pei-Zhi Liu, Hong-Ying Wen, Dong Tian

**Affiliations:** ^1^ Department of Thoracic Surgery, West China Hospital, Sichuan University, Chengdu, China; ^2^ Department of Cardiothoracic Intensive Care Unit, Affiliated Hospital of North Sichuan Medical College, Nanchong, China; ^3^ College of Medical Imaging, North Sichuan Medical College, Nanchong, China; ^4^ Department of Radiological Sciences, Graduate School of Biomedical Sciences, Nagasaki University, Nagasaki, Japan; ^5^ College of Clinical Medicine, North Sichuan Medical College, Nanchong, China; ^6^ Academician (Expert) Workstation, Affiliated Hospital of North Sichuan Medical College, Nanchong, China

**Keywords:** esophageal squamous cell carcinoma, recurrent laryngeal nerve, lymph node metastasis, diameter of lymph node, dynamic nomogram

## Abstract

**Background:**

Patients with esophageal squamous cell carcinoma (ESCC) are liable to develop recurrent laryngeal nerve (RLN) lymph node metastasis (LNM). We aimed to assess the predictive value of the long diameter (LD) and short diameter (SD) of RLN lymph node (LN) and construct a web-based dynamic nomogram for RLN LNM prediction.

**Methods:**

We reviewed 186 ESCC patients who underwent RLN LN dissection from January 2016 to December 2018 in the Affiliated Hospital of North Sichuan Medical College. Risk factors for left and right RLN LNM were determined by univariate and multivariate analyses. A web-based dynamic nomogram was constructed by using logistic regression. The performance was assessed by the area under the curve (AUC) and Brier score. Models were internally validated by performing five-fold cross-validation.

**Results:**

Patients who underwent left and right RLN LN dissection were categorized as left cohort (*n* = 132) and right cohort (*n* = 159), with RLN LNM rates of 15.9% (21/132) and 21.4% (34/159), respectively. The AUCs of the LD (SD) of RLN LN were 0.663 (0.688) in the left cohort and 0.696 (0.705) in the right cohort. The multivariate analysis showed that age, the SD of RLN LN, and clinical T stage were significant risk factors for left RLN LNM (all *P* < 0.05), while tumor location, the SD of RLN LN, and clinical T stage were significant risk factors for right RLN LNM (all *P* < 0.05). The dynamic nomograms showed reliable performance after five-fold cross-validation [(left (right), mean AUC: 0.814, range: 0.614–0.891 (0.775, range: 0.084–0.126); mean Brier score: 0.103, range: 0.084–0.126 (0.145, range: 0.105–0.206)], available at https://mpthtw.shinyapps.io/leftnomo/ and https://mpthtw.shinyapps.io/rightnomo/.

**Conclusion:**

The LD and SD of RLN LN are inadequate to predict RLN LNM accurately, but online dynamic nomograms by combined risk factors show better prediction performance and convenient clinical application.

## Introduction

Globally, the ratio of squamous cell cancers to adenocarcinomas has seen a reversal, but esophageal squamous cell cancer (ESCC) in China remains a dominant disease condition (1, 2). As a curative treatment, esophagectomy is accompanied by considerable postoperative morbidity and mortality (2). Recently, endoscopic submucosal dissection was found to be a better choice for removing superficial lesions, but this cannot be applied for involved lymph nodes (LNs) (3, 4). Therefore, significant efforts are necessary to improve the detection of early LN metastasis (LNM).

Located at the cervical base to the upper mediastinum, the recurrent laryngeal nerve (RLN) LN is one of the most common sites of LNM in thoracic ESCC (5), with reported incidences ranging from 18% to 63% (6). RLN LNM has been confirmed to be a strong predictor of poor prognosis in thoracic ESCC (7), with dismal 2- and 5-year survival rates of 28% and 10% (8). Additionally, some studies hypothesized that RLN LNs were the LNs for metastasis to cervical LNs (9), which could directly enter the lymphatic vessels of the supraclavicular LNs. However, a thorough and complicated dissection could increase the risk of postoperative complications such as RLN paralysis (10). Considering this situation, the detection of preoperative RLN LNM would be essential to the treatment strategy. However, to date, no satisfactory tool has been developed for making an accurate diagnosis of RLN LNM.

Previously, some studies reported that there might be a certain relationship between the diameter of RLN LNs and RLN LNM (11–13). However, the size overlap resulted in the poor performance during the process of distinguishing malignant nodes from benign ones (14). The predictive value of the length criteria of RLN LNs remains unsatisfactory. Several studies have built nomograms to predict RLN LNM in thoracic esophageal cancer but based on postoperative parameters (6, 15, 16), which may limit clinical application. Currently, most prediction models are still static and require manual calculations with low repeatability and poor intelligence. With the popularization of intelligent computers, the web-based nomogram offers much greater convenience. In this study, we aimed to assess the prediction competence of the long diameter (LD) and short diameter (SD) of RLN LN and construct web-based dynamic nomograms to predict the individual risk of left and right RLN LNM by preoperative parameters.

## Methods

### Patients

**Figure 1:**
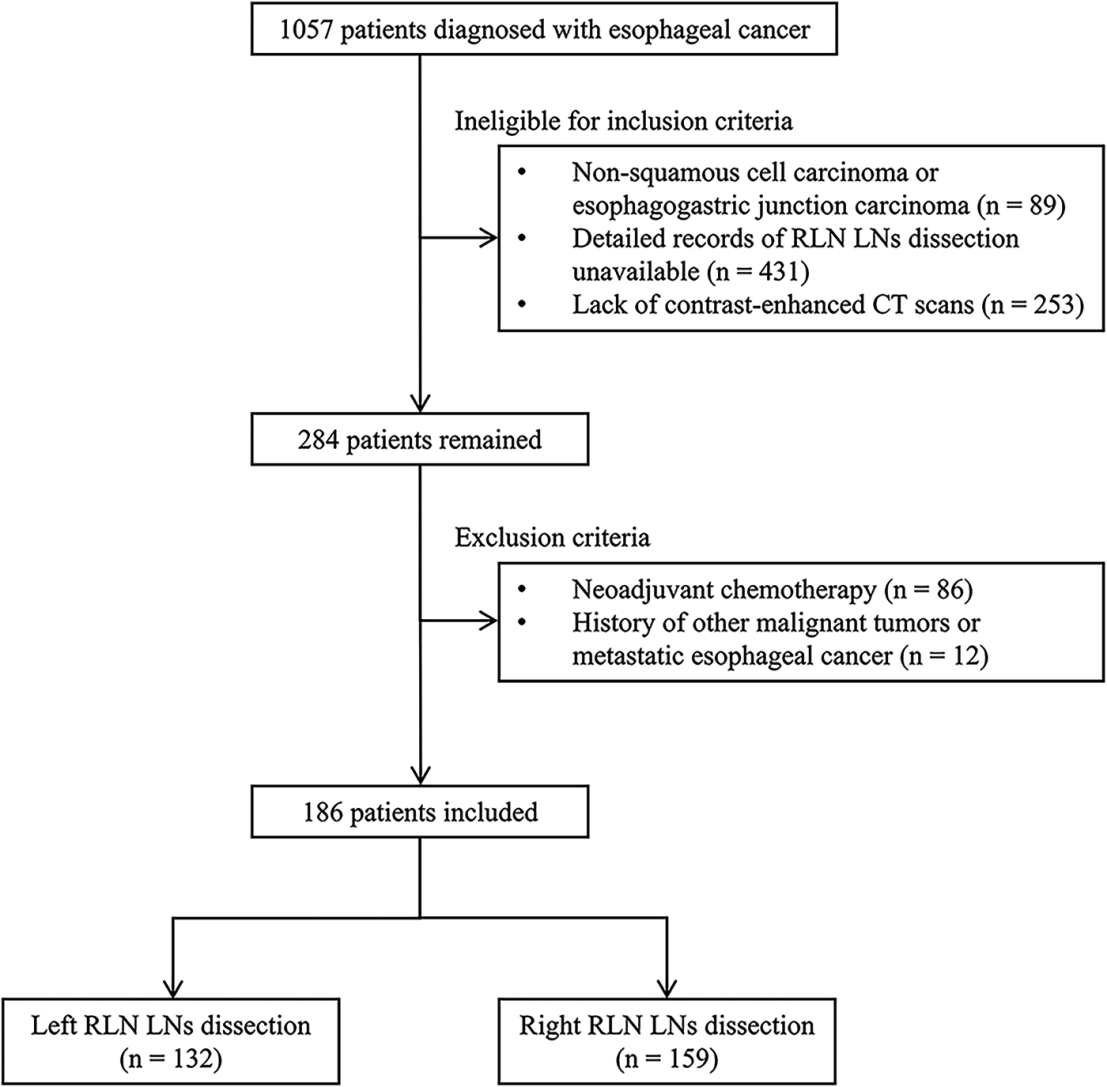
The selection process for including eligible patients in this study. RLN LNs, recurrent laryngeal nerve lymph nodes; CT, computed tomography.

The present study included patients with ESCC who underwent McKeown esophagectomy and RLN LN dissection (LND) in the Affiliated Hospital of North Sichuan Medical College between January 2016 and December 2018. The following criteria were used for the inclusion of patients in the current study: (a) pathologically confirmed ESCC, (b) patients with detailed records of RLN LND, and (c) contrast-enhanced computed tomography (CT) performed less than 2 weeks before surgical resection. The exclusion criteria were: (a) patients treated with neoadjuvant therapies (chemotherapy, immunotherapy, or radiation therapy) and (b) other histories of malignant tumors or metastatic esophageal cancer (Figure 1). Patients who underwent left or right RLN LND were categorized as left cohort or right cohort.

### Parameter measurements

The following clinical characteristics were reviewed: age, sex, tumor location, the LD and SD of RLN LN, clinical T (cT) stage, preoperative comorbidity, preoperative blood parameters of neutrophils, lymphocyte, hemoglobin, alanine transaminase, aspartate aminotransferase, albumin, high-density lipoprotein, low-density lipoprotein, and triglycerides. Within 2 weeks before surgery, all patients were scanned with contrast-enhanced CT from the neck to the abdomen. The LD and SD of RLN LN were mutually perpendicular and measured on the slice of maximum cross sections based on the single biggest LN. All images were anonymized and measured by using 3D slicer software (version 4.10.2, https://www.slicer.org). The measurement was completed by two thoracic radiologists, who reached a consensus about the diameter measurement. Both LD and SD were categorized into two groups at the optimal threshold by receiver operator characteristic (ROC) curves. The cT stage was evaluated by preoperative examinations based on the 8th edition of staging manual from the American Joint Committee on Cancer (AJCC) and the Union for International Cancer Control (UICC) (17).

### Development and validation of the dynamic nomograms

The risk factors for RLN LNM were determined by employing univariate analysis. Variables with a *P*-value <0.1 were entered into a multivariate logistic regression to determine the independent risk factors for predicting RLN LNM. The preoperative prediction model was established by using the independent risk factors. The dynamic nomogram was generated as an online scoring system to visualize the model based on risk factors. The area under the ROC curves (AUCs) were used to evaluate the discrimination of this nomogram. The Brier score is a proper scoring rule of calibration, ranging from 0 to 1, with 0 representing the best possible calibration. The calibration curve was also plotted to graphically assess the calibration. The decision curve analysis (DCA) was performed to evaluate the net benefits at each threshold probability of the nomogram. Model performance was internally validated by conducting five-fold cross-validation. Out of five subsamples, a single subsample was retained as the validation data for testing, and the other four subsamples were used for training. With five-time repetitions, each set exactly served as the validation data for one repetition. The AUCs and Brier scores from the folds will be averaged to produce a single estimation for assessing reliability.

### Statistical analysis

The statistical analysis was executed by using IBM SPSS Statistics (version 22.0 Inc., Chicago, IL, USA) and the R language (version 3.6.3, Vienna, Austria). Continuous variables were represented as mean with standard deviation and categorical variables were summarized by way of count and percentage. The odds ratio (OR) and 95% confidence interval (CI) were figured. To avoid potential multicollinearity, the Kendall rank correlation analysis was performed between the LD and the SD of RLN LN since both of these were the morphological features of nodes. A *P*-value <0.05 was regarded as statistically significant.

## Results

### Patient characteristics

The demographic and preoperative characteristics of ESCC patients are summarized in Table 1. Out of 186 patients who were included in this study, 132 were in the left cohort and 159 were in the right cohort. The incidences of RLN LNM in the left and right cohorts were 15.9% (21/132) and 21.4% (34/159), respectively. There were 46 patients ≤60 years and 86 patients >60 years in the left cohort, with correspondingly 42 and 117 patients in the other cohort. As for tumor location, the number of patients with upper, middle, and lower lesions were 24, 78, and 30 in the left cohort and 19, 94, and 46 in the right cohort. The mean LD and SD of left RLN LN were 5.50 ± 2.13, 4.10 ± 1.58 mm and those of right RLN LN were 6.43 ± 2.53, 4.68 ± 1.85 mm.

**Table 1:** Summary of clinical characteristics in the study population.

Characteristics	Left cohort (*n* = 132)	Right cohort (*n* = 159)
Age (year)		
≤60	46 (34.8%)	42 (26.4%)
>60	86 (65.2%)	117 (73.6%)
Sex		
Male	90 (68.2%)	106 (66.7%)
Female	42 (31.8%)	53 (33.3%)
Tumor location		
Upper	24 (18.2%)	19 (11.9%)
Middle	78 (59.1%)	94 (59.1%)
Lower	30 (22.7%)	46 (28.9%)
LD of RLN LN (mm)	5.50 ± 2.13	6.43 ± 2.53
SD of RLN LN (mm)	4.10 ± 1.58	4.68 ± 1.85
cT stage		
cT1	32 (24.2%)	52 (32.7%)
cT2	49 (37.1%)	66 (41.5%)
cT3	48 (36.4%)	39 (24.5%)
cT4	3 (2.3%)	2 (1.3%)
Preoperative complications		
Absent	39 (29.5%)	60 (37.7%)
Present	93 (70.5%)	99 (62.3%)
Preoperative neutrophils	3.96 ± 2.12	4.19 ± 2.22
Preoperative lymphocyte	1.76 ± 1.43	1.62 ± 1.32
Preoperative hemoglobin	128.04 ± 15.64	125.75 ± 15.10
Preoperative ALT	24.51 ± 70.71	20.61 ± 13.12
Preoperative AST	25.03 ± 39.67	21.68 ± 11.39
Preoperative albumin	39.34 ± 3.38	39.22 ± 3.54
Preoperative high-density lipoprotein	1.30 ± 0.31	1.25 ± 0.33
Preoperative low-density lipoprotein	2.58 ± 0.68	2.56 ± 0.67
Preoperative triglycerides	1.32 ± 0.80	1.39 ± 0.74

RLN LN, recurrent laryngeal nerve lymph node; LD, long diameter; SD, short diameter; cT stage, clinical T stage. Continuous variables were represented as mean with standard deviation and categorical variables were summarized by count and percentage.

### Predictive values of the LD and SD of RLN LN

**Figure 2:**
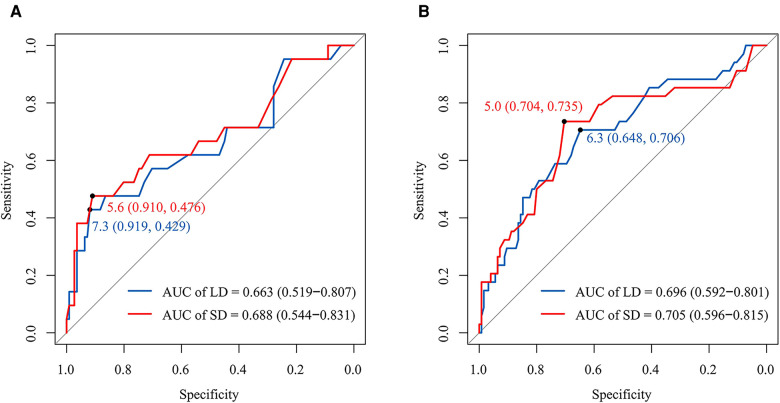
The receiver operator characteristic curves for the LD and SD of RLN LN in the left and right cohorts, respectively. The AUCs of the LD and SD of left RLN LN were 0.663 (95% CI, 0.519–0.807) and 0.688 (95% CI, 0.544-0.831), respectively. The optimal thresholds of the LD and SD of left RLN LN for RLN LNM were at 7.3 mm (sensitivity and specificity: 42.9% and 91.9%) and 5.6 mm (sensitivity and specificity: 47.6% and 91.0%) (**A**). The AUCs of the LD and SD of right RLN LN were 0.696 (95% CI, 0.592–0.801) and 0.705 (95% CI, 0.596–0.815), respectively. The optimal thresholds of the LD and SD of right RLN LN for RLN LNM were at 6.3 mm (sensitivity and specificity: 70.6% and 64.8%) and 5.0 mm (sensitivity and specificity: 73.5% and 70.4%) (**B**). LD, long diameter; SD, short diameter; AUC, the area under the curve; RLN LNs, recurrent laryngeal nerve lymph nodes.

In the left cohort, the AUCs of the LD and SD of RLN LN were 0.663 (95 CI%, 0.519–0.807) and 0.688 (95 CI%, 0.544–0.831), with optimal thresholds of 7.3 and 5.6 mm. The specificity and sensitivity at the optimal threshold of LD (SD) were 91.9% (91.0%) and 42.9% (47.6%), respectively (Figure 2A). In the right cohort, the AUCs of the LD and SD of RLN LN were 0.696 (95 CI%, 0.592–0.801) and 0.705 (95 CI%, 0.596–0.815), respectively. The cutoff values of LD and SD were 6.3 and 5.0 mm. The specificity and sensitivity at the optimal threshold of LD (SD) were 64.8% (70.4%) and 70.6% (73.5%), respectively (Figure 2B).

### Risk factors for RLN LNM

In the left cohort, the univariate analysis showed that age, the LD of left RLN LN, the SD of left RLN LN, and cT stage were significantly associated with RLN LNM. The Kendall rank correlation analysis coefficient between the LD and the SD of left RLN LN was 0.694 (*P* < 0.001), which indicated a strong direct correlation between two variables. To avoid potential multicollinearity between LD and SD, only the SD of left RLN LN was entered into the subsequent multivariate analysis owing to a higher AUC [LD, 0.663 vs. SD, 0.688]. The multivariate logistic regression analysis showed that age (OR = 0.286, 95% CI, 0.088–0.930, *P* = 0.038), the SD of left RLN LN (OR = 14.780, 95% CI, 3.929–55.601, *P* < 0.001), and cT stage (OR = 3.422, 95% CI, 1.507–7.772, *P* = 0.003) were independent risk factors for left RLN LNM (Table 2). The univariate analysis showed tumor location, the LD of right RLN LN, the SD of right RLN LN, and cT stage were significantly associated with right RLN LNM. The Kendall rank correlation analysis coefficient was 0.677 (*P* < 0.001), suggesting that LD was closely related to SD. Likewise, only the SD of right RLN LN was set into the multivariate analysis because of the higher AUC to avoid potential multicollinearity [LD, 0.696 vs. SD, 0.705]. The multivariate logistic regression analysis showed that tumor location (OR = 0.250, 95% CI, 0.107–0.588, *P* < 0.001), the SD of right RLN LN (OR = 10.430, 95% CI, 3.770–28.850, *P* < 0.001), and cT stage (OR = 2.646, 95% CI, 1.380–5.703, *P* = 0.003) were independent risk factors for RLN LNM (Table 3).

**Table 2:** Univariate and multivariate analyses for risk factors of left RLN LNM.

Parameters	Univariate analysis			Multivariate analysis		
	OR	95% CI	*P*	OR	95% CI	*P*
Age (≤60/>60)	0.331	0.128–0.860	0.023*	0.286	0.088–0.930	0.038*
Sex (male/female)	1.086	0.403–2.927	0.871			
Tumor location^¶^	1.537	0.729–3.241	0.259			
LD of RLN LN (<7.30/≥7.30 mm)	8.500	2.828–25.552	<0.001*			
SD of RLN LN (<5.60/≥5.60 mm)	9.182	3.134–26.902	<0.001*	14.780	3.929–55.601	<0.001*
cT stage^#^	2.733	1.381–5.410	0.004*	3.422	1.507–7.772	0.003*
Preoperative complications (absent/present)	1.957	0.613–6.246	0.257			
Preoperative neutrophils	0.764	0.541–1.077	0.125			
Preoperative lymphocyte	0.972	0.676–1.397	0.878			
Preoperative hemoglobin	1.003	0.973–1.033	0.865			
Preoperative ALT	1.006	0.994–1.019	0.328			
Preoperative AST	1.012	0.988–1.036	0.325			
Preoperative albumin	1.023	0.891–1.174	0.750			
Preoperative high-density lipoprotein	5.336	1.233–23.086	0.025*	3.821	0.630–23.168	0.145
Preoperative low-density lipoprotein	1.445	0.740–2.821	0.281			
Preoperative triglycerides	0.777	0.373–1.620	0.501			

RLN LNM, recurrent laryngeal nerve lymph node metastasis; RLN LN, recurrent laryngeal nerve lymph node; LD, long diameter; SD, short diameter; OR, odds ratio; CI, confidence interval; cT stage, clinical T stage; ALT, alanine transaminase; AST, aspartate aminotransferase.

^¶^ Tumor location was classified into upper, middle, and lower parts, with assigned values of 1, 2, and 3, respectively; *P < 0.05; ^#^cT stage consisted of four stages, cT1, cT2, cT3, and cT4, with assigned values of 1, 2, 3, and 4, respectively.

**Table 3:** Univariate and multivariate analyses for risk factors of right RLN LNM.

Parameters	Univariate analysis			Multivariate analysis		
	OR	95% CI	*P*	OR	95% CI	*P*
Age (≤60/>60)	0.826	0.357–1.913	0.655			
Sex (male/female)	0.945	0.421–2.122	0.891			
Tumor location^¶^	0.457	0.240–0.869	0.017*	0.250	0.107–0.588	0.001*
LD of RLN LN (<6.30/≥6.30 mm)	4.418	1.938–10.071	<0.001*			
SD of RLN LN (<5.00/≥5.00 mm)	5.708	2.485–13.112	<0.001*	10.430	3.770–28.850	<0.001*
cT stage^#^	1.715	1.051–2.800	0.031*	2.646	1.380–5.703	0.003*
Preoperative complications (absent/present)	1.143	0.518–2.519	0.741			
Preoperative neutrophils	1.002	0.845–1.188	0.980			
Preoperative lymphocyte	0.699	0.372–1.311	0.264			
Preoperative hemoglobin	1.003	0.978–1.028	0.842			
Preoperative ALT	0.999	0.970–1.029	0.935			
Preoperative AST	0.995	0.960–1.031	0.782			
Preoperative albumin	1.047	0.940–1.167	0.405			
Preoperative high-density lipoprotein	2.879	0.930–8.910	0.067	3.907	0.923–16.532	0.064
Preoperative low-density lipoprotein	1.676	0.946–2.969	0.077	2.037	0.984–4.220	0.055
Preoperative triglycerides	0.790	0.435–1.434	0.438			

RLN LNM, recurrent laryngeal nerve lymph node metastasis; RLN LN, recurrent laryngeal nerve lymph node; LD, long diameter; SD, short diameter; OR, odds ratio; CI, confidence interval; cT stage, clinical T stage; ALT, alanine transaminase; AST, aspartate aminotransferase.

^¶^ Tumor location was classified into upper, middle, and lower parts, with assigned values of 1, 2, and 3, respectively; *P < 0.05; ^#^cT stage consisted of four stages, cT1, cT2, cT3, and cT4, with assigned values of 1, 2, 3, and 4, respectively.

### Development of the nomograms

**Figure 3:**
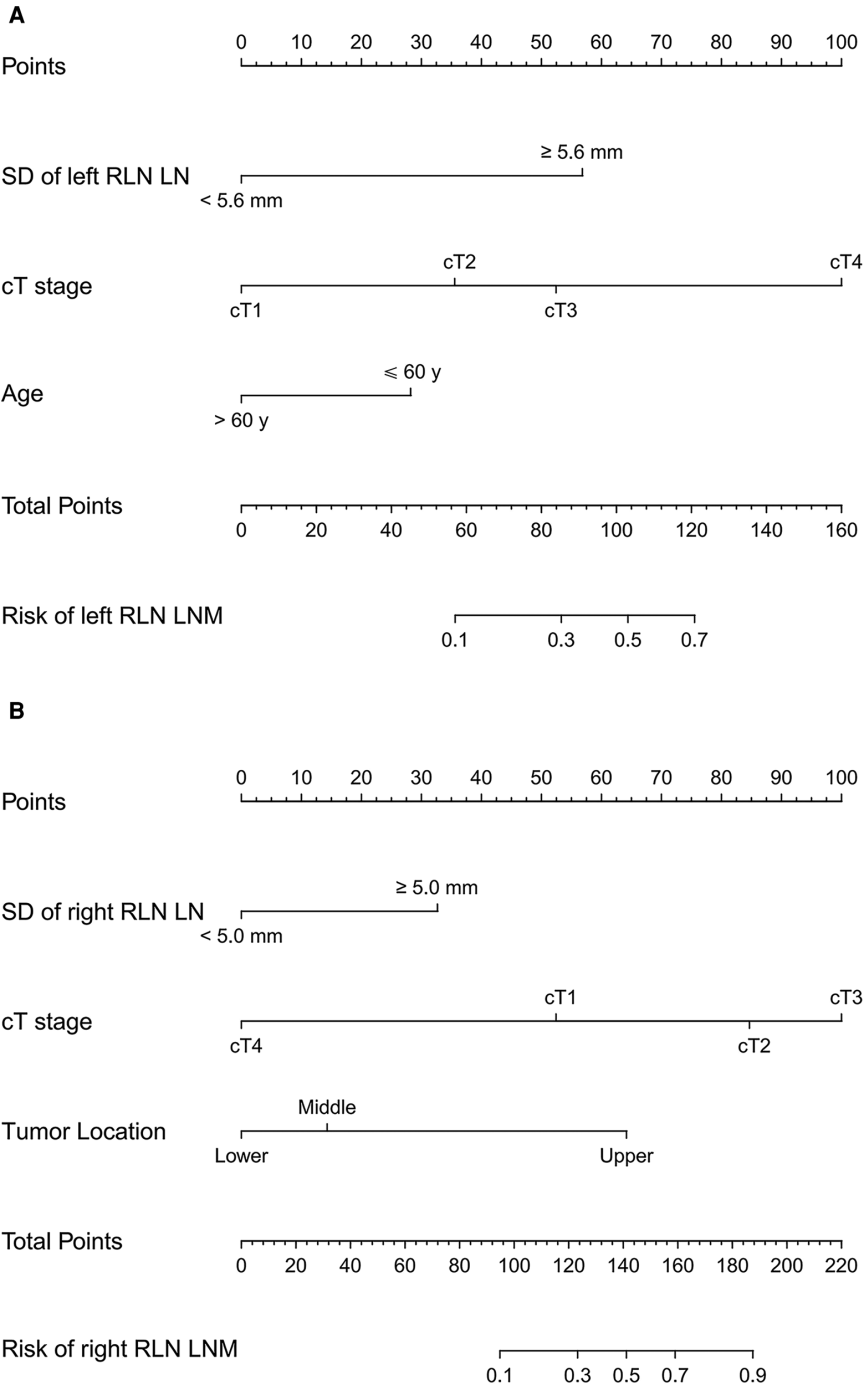
Traditional nomograms for left and right RLN LNM prediction. The nomogram for left RLN LNM incorporated the SD of left RLN LN, cT stage, and age (**A**). The nomogram for right RLN LNM incorporated the SD of right RLN LN, cT stage, and tumor location (**B**). RLN LNM, recurrent laryngeal nerve lymph node metastasis; RLN LN, recurrent laryngeal nerve lymph node; SD, short diameter; cT stage, clinical T stage.

**Figure 4:**
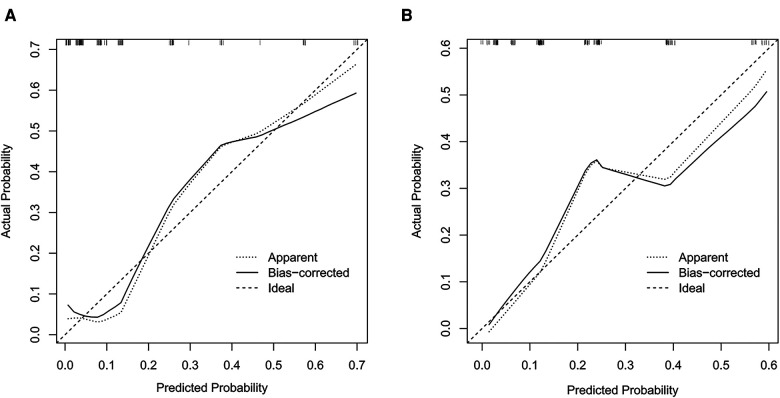
Calibration curves of the nomograms for left and right RLN LNM prediction. The calibration curves demonstrated that the predicted probability by nomogram was closely aligned with the actual estimates in both left (**A**) and right (**B**) cohorts, indicating that there was good agreement. RLN LNM, recurrent laryngeal nerve lymph node metastasis.

**Figure 5:**
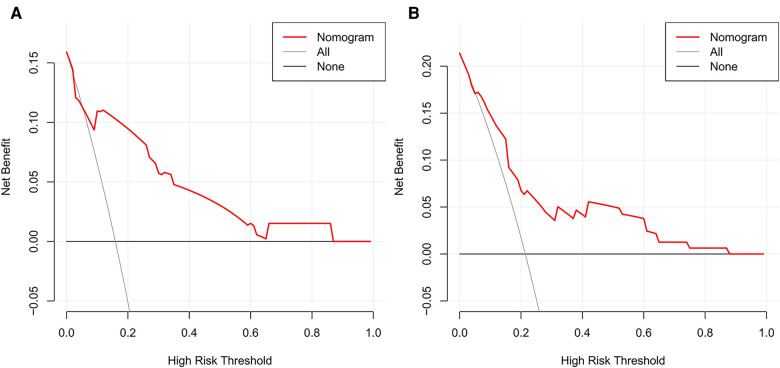
Decision curve analysis of the nomograms for left and right RLN LNM prediction. DCA plots revealed that the nomogram had good net benefits in both left (**A**) and right (**B**) cohorts. RLN LNM, recurrent laryngeal nerve lymph node metastasis. DCA, decision curve analysis.

The prediction models were established according to multivariate logistic regression analysis (Figure 3A,B). In the left cohort, the age, the SD of left RLN LN, and cT stage were included in the dynamic nomogram. The AUCs and Brier score of the nomogram were 0.855 (95% CI, 0.759–0.950) and 0.093, respectively, suggesting a reliable performance in prediction. In the right cohort, the dynamic nomogram was constructed with the SD of right RLN LN, cT stage, and tumor location, with an AUC and Brier score of 0.789 (95% CI, 0.711–0.867) and 0.135, respectively, which indicated satisfactory competence in predicting right RLN LNM. The calibration curve showed the dots close to the ideal line in both cohorts, indicating that the nomograms were well-calibrated (Figures 4A,B). DCA curves, with threshold probabilities and net benefits on the horizontal and vertical axes, showed that both nomograms yielded clinical benefits (Figures 5A,B).

### Validation of the nomograms

After five-fold cross-validation, the mean AUC and Brier score of the nomogram were 0.814 (range: 0.659–0.943) and 0.103 (range: 0.084–0.126) in the left cohort, still indicating solid discrimination and calibration. In the other cohort, the mean AUC and Brier score of the nomogram were 0.775 (range: 0.614–0.891) and 0.145 (range: 0.105–0.206), respectively, suggesting satisfactory performance for RLN LNM prediction.

### Examples of the dynamic nomograms

**Figure 6:**
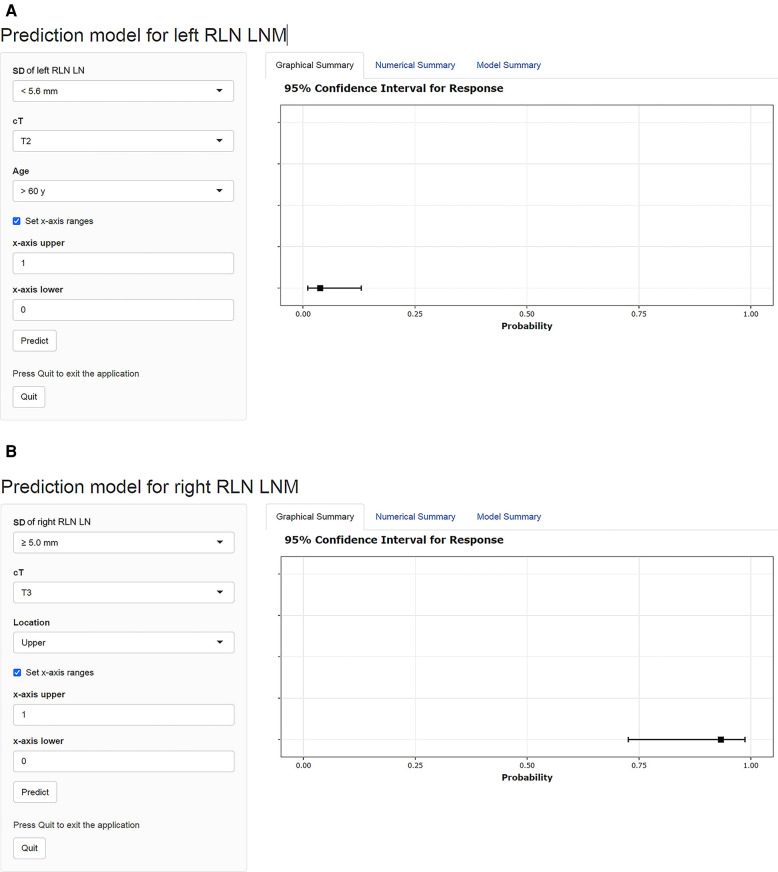
Examples of the dynamic nomograms for left and right RLN LNM prediction. The probability of RLN LNM is shown on the right side of the screen after each parameter is entered on the left input field. The risk of left RLN LNM was at 3.8% for a patient with an SD of left RLN LN <5.6 mm, cT2 stage, age >60 years (**A**). A patient diagnosed with an SD of right RLN LN ≥ 5.6 mm, cT3 stage, and upper lesion might suffer right RLN LNM at a risk of 93.3% (**B**). RLN LNM, recurrent laryngeal nerve lymph node metastasis; RLN LN, recurrent laryngeal nerve lymph node; SD, short diameter; cT, clinical T stage.

The dynamic nomograms were generated as online scoring systems in both cohorts, which were available at https://mpthtw.shinyapps.io/leftnomo/ and https://mpthtw.shinyapps.io/rightnomo/ for clinical use and future validation. For example, the risk of left RLN LNM was at 3.8% for a patient with the SD of left RLN LN <5.6 mm, cT2 stage, age >60 years (Figure 6A). Another example is that a patient considered the SD of right RLN LN ≥ 5.6 mm, cT3 stage, and upper lesion might suffer right RLN LNM at a risk of 93.3% (Figure 6B).

## Discussion

The significance of this study is to develop a preoperative diagnostic tool to predict the individual risk for RLN LNM in ESCC with reliable accuracy. We found that the predictive value of the LD and SD of RLN LN were inadequate for LNM detection. According to multivariate analysis, age, the SD of left RLN LN, and cT stage were independent risk factors for left RLN LNM, while the other cohort showed tumor location, the SD of RLN LN, and cT stage were independent risk factors for right RLN LNM. Based on the above, we established and validated a web-based dynamic nomogram by using independent risk factors and with robust prediction performance.

There is a trade-off between the extended lymphadenectomy with potential survival benefit and two-field dissection with decreased postoperative morbidity. Undoubtedly, both groups of patients who underwent unilateral and bilateral RLN LND had significantly better disease-free survival outcomes and overall survival outcomes than those without RLN LND (18). However, a thorough RLN LND increases the risk of nerve injury and may lead to the early postoperative complications of aspiration and respiratory insufficiency, thus sacrificing the quality of life. Based on LN status, surgeons could discourage relapse from insufficient dissection among susceptible candidates and prevent potential complications from extended surgery for those without metastasis. To sum up, the identification of preoperative RLN LNM would be essential to the treatment decision.

As an affordable examination, CT is readily available for most patients before treatment, with a sensitivity of 50% (range: 41%–60%) and an accuracy of 63% (range: 53%–72%) (2), which depends on the size definition for abnormally enlarged LNs. In this research, the AUCs of LD (SD) based on CT were 0.663 (0.688) in the left cohort and 0.696 (0.705) in the right cohort, indicating relatively low discrimination. This inaccurate prediction might be explained by the fact that typically sized LNs contained metastatic deposits or got inflamed, leading to enlargement, which was hard to recognize because of their large quantity and tiny distinction. The cutoff value of the LD (SD) of RLN LN in the left and right cohorts were 7.3 (5.6) and 6.0 (5.0) mm, respectively. The optimal threshold of Chen et al. (19) and Li Bin et al. (20) were 5.5 mm for left RLN LN and 6.5 mm of SD for right RLN LN, which were close to our result. However, it was implied that the size criteria had limited confidence in predicting RLN LNM regardless of the SD or LD of RLN LN in the current study. RLN LNM could not be determined by the size evaluated by CT alone.

Previous studies have outlined various risk factors associated with RLN LNM for ESCC, including tumor location, primary tumor differentiation, tumor invasion depth, and so on (21–23). In this research, additional clinical blood parameters were introduced into the logistic regression to predict the probability of RLN LNM as accurately as possible. Remarkably, we identified separate risk factors for the left and right cohorts according to multivariate analysis, which might be caused by the particularity of the left and right RLN LNs. Similarly, Liu et al. (15) and Zhang et al. (6) also determined different risk factors for left and right cohorts due to the anatomical differences in their studies. In the current study, age was presented to be a significant protective factor in the regression analysis for left RLN LNM, indicating that a higher age leads to a lower risk for metastasis. However, this conclusion was not suitable for right RLN LNs in our study. The discrepancy might be caused by the sex ratio between cohorts. Although many trials have reported that senior age was a risk factor for LNM (6, 24), Yu et al. (16) reported that age <56 years was significantly associated with a higher risk of RLN LNM, which was consistent with our conclusion. Thus, the influence of age on RLN LNM remained controversial, and future studies with larger samples will be crucial to answer this question.

Advanced cT stage is one of the influential contributors to the risk of bilateral RLN LNM. In the left cohort, the rates of RLN LNM in patients with cT1, cT2, cT3, and cT4 stages were 3.1%, 14.3%, 22.9%, and 66.7%, respectively. In the right cohort, the rates were 11.5%, 22.7%, and 33.0%, respectively, and there was no patient with cT4 stage. With the deeper tumor invasion, we could see an upward trend in the incidences of RLN LNM, which was consistent with the studies of Ye et al. (23) and Betancourt et al. (25). Anatomically, without the serosal layer, the esophageal wall was penetrated by rich lymphatic vessels in a transverse and longitudinal way (26). The deeper the infiltration into the submucosa, the easier malignant cells can be transferred by longitudinal vessels, causing the high probability of RLN LNM in advanced stages (27). However, some skip metastasis can be present only in mucosa invasion (28).

Existing definite evidence showed that the incidences of bilateral RLN LNM for upper tumors were the highest (29). This might be associated with the fact that specific tumor location was correspondent to the distribution pattern of regional LNs. The lesions located in the upper segment were prone to transfer to RLN LNs through the longitudinal lymphatic network. According to multivariate analysis in our research, the tumor location was considered a significant risk factor for right RLN LNM. Likewise, this finding was supported by the studies of Yu et al. (16) and Zhang et al. (18), which also showed that RLN LNM was positively associated with upper and middle tumors. However, this trend did not occur in the left cohort, where RLN LNM was associated with lower lesions, not statistically. This might be due to the different proportions of tumor locations in our study.

Previously, Liu et al. (15) presented a prediction model based on clinicopathological characteristics for RLN LNM, but postoperative variables limited its clinical value. Zhang et al. (6) developed a nomogram and recursive partitioning analysis but these lacked validation information. In this study, we applied significant preoperative variables to generate a dynamic nomogram. The prediction model performed satisfactorily for the prediction of left and right RLN LNM. The calibration curves and DCA also confirmed this effective clinical performance. In addition, we conducted five-fold cross-validation to avoid overfitting. The comparable results after validation also indicated that the nomograms were robust in quantifying an individual risk for RLN LNM. Our prediction model can straightforwardly figure out the risk of RLN LNM through online tools, which can avoid the computational errors associated with traditional nomograms. The probability of RLN LNM is automatically exhibited on the right side of the screen after each parameter is set on the left side of the sketch map, without manual calculations. In addition, the simple and easy interface is more convenient for clinicians to make a treatment choice for an individual patient.

### Limitations

Several limitations exist in this study. First, it was a retrospective study with a small sample on single-center data and thus bias was inevitable. Strict inclusion and exclusion criteria were applied and data were processed by uniform definitions to control selection bias. Second, since RLN LNs were clustered along the nerves, the surgeons could not visualize the position of LNs after removal, which made it impossible to match those identified on CT and surgically excised tissues. Therefore, we could assess the ability of only the largest LNs rather than individual nodes. Third, only a small proportion of patients received PET/CT scans before the operation, which were inadequate for us to explore the predictive value of PET/CT for RLN LNM. Lastly, the validation process was conducted by using five-fold cross-validation rather than an independent validation set because of the small sample size. The division of the independent validation set will sacrifice the sample size of the training set, which will degrade the performance of predicting models. Despite its inability to perform comprehensive validation, the five-fold cross-validation method is eligible enough to verify the predictive ability of the nomogram. In conclusion, further studies with large samples in multicenter organizations are warranted.

## Conclusion

The LD and SD of RLN LN are inadequate to predict RLN LNM accurately. The dynamic nomograms based on risk factors show satisfactory performance for RLN LNM prediction. Future studies with multicenter data and large sample populations will be crucial to verify this model.

## Data Availability

The raw data supporting the conclusions of this article will be made available by the authors, without undue reservation.

## References

[1] Tramontano AC Nipp R Mercaldo ND Kong CY Schrag D Hur C. Survival disparities by race and ethnicity in early esophageal cancer. Dig Dis Sci. (2018) 63(11):2880–8. 10.1007/s10620-018-5238-6 30109578 PMC6738563

[2] Chen H Zhou X Tang X Li S Zhang G. Prediction of lymph node metastasis in superficial esophageal cancer using a pattern recognition neural network. Cancer Manag Res. (2020) 12:12249–58. 10.2147/cmar.S270316 33273861 PMC7707435

[3] Jacobs M Macefield RC Elbers RG Sitnikova K Korfage IJ Smets EM Meta-analysis shows clinically relevant and long-lasting deterioration in health-related quality of life after esophageal cancer surgery. Qual Life Res. (2014) 23(4):1097–115. 10.1007/s11136-013-0545-z 24129668

[4] Aadam AA Abe S. Endoscopic submucosal dissection for superficial esophageal cancer. Dis Esophagus. (2018) 31(7). 10.1093/dote/doy021 29982386

[5] Wu J Chen QX Zhou XM Mao WM Krasna MJ. Does recurrent laryngeal nerve lymph node metastasis really affect the prognosis in node-positive patients with squamous cell carcinoma of the middle thoracic esophagus? BMC Surg. (2014) 14:43. 10.1186/1471-2482-14-43 25016483 PMC4105105

[6] Zhang G Li Y Wang Q Zheng H Yuan L Gao Z Development of a prediction model for the risk of recurrent laryngeal nerve lymph node metastasis in thoracolaparoscopic esophagectomy with cervical anastomosis. Ann Transl Med. (2021) 9(12):990. 10.21037/atm-21-2374 34277790 PMC8267307

[7] Malassagne B Tiret E Duprez D Coste J de Sigalony JP Parc R. Prognostic value of thoracic recurrent nerve nodal involvement in esophageal squamous cell carcinoma. J Am Coll Surg. (1997) 185(3):244–9. 10.1016/s1072-7515(97)00046-x 9291401

[8] Baba M Aikou T Yoshinaka H Natsugoe S Fukumoto T Shimazu H Long-term results of subtotal esophagectomy with three-field lymphadenectomy for carcinoma of the thoracic esophagus. Ann Surg. (1994) 219(3):310–16. 10.1097/00000658-199403000-00012 8147613 PMC1243140

[9] Ueda Y Shiozaki A Itoi H Okamoto K Fujiwara H Ichikawa D Intraoperative pathological investigation of recurrent nerve nodal metastasis can guide the decision whether to perform cervical lymph node dissection in thoracic esophageal cancer. Oncol Rep. (2006) 16(5):1061–6. 10.3892/or.16.5.1061 17016593

[10] Hong TH Kim HK Lee G Shin S Cho JH Choi YS Role of recurrent laryngeal nerve lymph node dissection in surgery of early-stage esophageal squamous cell carcinoma. Ann Surg Oncol. (2022) 29(1):627–39. 10.1245/s10434-021-10757-w 34480274

[11] Yamaoka Y Kinugasa Y Shiomi A Yamaguchi T Kagawa H Yamakawa Y The distribution of lymph node metastases and their size in colon cancer. Langenbeck's Arch Surg. (2017) 402(8):1213–21. 10.1007/s00423-017-1628-z 28983781

[12] Liu J Wang Z Shao H Qu D Liu J Yao L. Improving CT detection sensitivity for nodal metastases in oesophageal cancer with combination of smaller size and lymph node axial ratio. Eur Radiol. (2018) 28(1):188–95. 10.1007/s00330-017-4935-4 28677059

[13] Gore RM. Upper gastrointestinal tract tumours: diagnosis and staging strategies. Cancer Imaging. (2005) 5(1):95–8. 10.1102/1470-7330.2005.0020 16154827 PMC1665231

[14] Moug SJ Saldanha JD McGregor JR Balsitis M Diament RH. Positive lymph node retrieval ratio optimises patient staging in colorectal cancer. Br J Cancer. (2009) 100(10):1530–3. 10.1038/sj.bjc.6605049 19401684 PMC2696755

[15] Liu Y Zou ZQ Xiao J Zhang M Yuan L Zhao XG. A nomogram prediction model for recurrent laryngeal nerve lymph node metastasis in thoracic oesophageal squamous cell carcinoma. J Thorac Dis. (2019) 11(7):2868–77. 10.21037/jtd.2019.06.46 31463116 PMC6688001

[16] Yu S Lin J Chen C Lin J Han Z Lin W Recurrent laryngeal nerve lymph node dissection may not be suitable for all early stage esophageal squamous cell carcinoma patients: an 8-year experience. J Thorac Dis. (2016) 8(10):2803–12. 10.21037/jtd.2016.10.34 27867556 PMC5107489

[17] Escrig Sos J Gómez Quiles L Maiocchi K. The 8th edition of the AJCC-TNM classification: new contributions to the staging of esophagogastric junction cancer. Cir Esp. (2019) 97(8):432–7. 10.1016/j.ciresp.2019.03.006 31029372

[18] Zhang S Liu Q Li B Jia M Cai X Yang W Clinical significance and outcomes of bilateral and unilateral recurrent laryngeal nerve lymph node dissection in esophageal squamous cell carcinoma: a large-scale retrospective cohort study. Cancer Med. (2022) 11(7):1617–29. 10.1002/cam4.4399 PMC8986140 35174645

[19] Chen C Ma Z Shang X Duan X Yue J Jiang H. Risk factors for lymph node metastasis of the left recurrent laryngeal nerve in patients with esophageal squamous cell carcinoma. Ann Transl Med. (2021) 9(6):476. 10.21037/atm-21-377 PMC8039656 33850873

[20] Li B Li B Jiang H Yang Y Zhang X Su Y The value of enhanced CT scanning for predicting lymph node metastasis along the right recurrent laryngeal nerve in esophageal squamous cell carcinoma. Ann Transl Med. (2020) 8(24):1632. 10.21037/atm-20-4991 33490144 PMC7812183

[21] Hsu P Huang C Hsieh C Wu Y Hsu W. Role of right upper mediastinal lymph node metastasis in patients with esophageal squamous cell carcinoma after tri-incisional esophagectomies. Surgery. (2014) 156(5):1269–77. 10.1016/j.surg.2014.05.007 24953277

[22] Yan HJ Mao WJ Yu RX Jiang KY Huang H Zong ZD Preoperative clinical characteristics predict recurrent laryngeal nerve lymph node metastasis and overall survival in esophageal squamous cell carcinoma: a retrospective study with external validation. Front Oncol. (2022) 12:859952. 10.3389/fonc.2022.859952 PMC9008727 35433473

[23] Ye K Xu J Sun Y Lin J Zheng Z. Characteristics and clinical significance of lymph node metastases near the recurrent laryngeal nerve from thoracic esophageal carcinoma. Genet Mol Res. (2014) 13(3):6411–19. 10.4238/2014.August.25.4 25158259

[24] Zheng Y Wang Z Wang F Huang Q Liu S. Proposed modifications of supraclavicular lymph node metastasis in the esophageal squamous cell carcinoma staging system for improved survival stratification. Oncotarget. (2017) 8(25):41563–71. 10.18632/oncotarget.14892 28147340 PMC5522297

[25] Betancourt Cuellar S Sabloff B Carter B Benveniste M Correa A Maru D Early clinical esophageal adenocarcinoma (cT1): utility of CT in regional nodal metastasis detection and can the clinical accuracy be improved? Eur J Radiol. (2017) 88:56–60. 10.1016/j.ejrad.2017.01.001 28189209

[26] Suda K Ishida Y Kawamura Y Inaba K Kanaya S Teramukai S Robot-assisted thoracoscopic lymphadenectomy along the left recurrent laryngeal nerve for esophageal squamous cell carcinoma in the prone position: technical report and short-term outcomes. World J Surg. (2012) 36(7):1608–16. 10.1007/s00268-012-1538-8 22392356

[27] Wang A Lu L Fan J Wang S Chen X. Lymph node metastatic patterns and its clinical significance for thoracic superficial esophageal squamous cell carcinoma. J Cardiothorac Surg. (2020) 15(1):262. 10.1186/s13019-020-01302-z 32958015 PMC7507729

[28] Duan XF Shang XB Tang P Jiang HJ Gong L Yue J Lymph node metastasis and prognostic factors for T1 esophageal cancer. Zhonghua wai ke za zhi [Chinese Journal of Surgery]. (2017) 55(9):690–5. 10.3760/cma.j.issn.0529-5815.2017.09.010 28870055

[29] Tachimori Y Ozawa S Numasaki H Matsubara H Shinoda M Toh Y Efficacy of lymph node dissection by node zones according to tumor location for esophageal squamous cell carcinoma. Esophagus. (2016) 13:1–7. 10.1007/s10388-015-0515-3 26752982 PMC4698372

